# Blistering Distal Dactylitis Caused by *Acinetobacter lwoffii* in an Immunocompetent Patient: A Case Report

**DOI:** 10.3390/reports9030241

**Published:** 2026-07-27

**Authors:** Ajlan Alajlani, Nouf Almagushi, Fahad Almuhaymizi, Ruaa Alharithy

**Affiliations:** 1 General Directorate of Prisons’ Health, Ministry of Interior, Riyadh 13321, Saudi Arabia; 2 College of Medicine, King Saud bin Abdulaziz University for Health and Sciences, Riyadh 11481, Saudi Arabia; 3 Department of Dermatology, Security Force Hospital, Riyadh 12623, Saudi Arabia; 4 Department of Dermatology, Princess Nourabent Abdulrahman University, Riyadh 11671, Saudi Arabia; 5 Department of Dermatology, Security Forces Hospital, Riyadh 11564, Saudi Arabia

**Keywords:** blistering distal dactylitis, *Acinetobacter lwoffii*, bullous dermatoses

## Abstract

**Background and Clinical Significance:** Blistering distal dactylitis is a localized infection of the distal phalanx, most commonly affecting children and adolescents and typically caused by group A β-hemolytic *Streptococcus*. Infections due to atypical organisms are rare. Involvement of atypical organisms such as *Acinetobacter lwoffii* is extremely rare; **Case Presentation:** We report an 18-month-old boy presenting with hemorrhagic crustation over bilateral big toes for 2 weeks; wound culture swab revealed *Acinetobacter lwoffii*. Patient was managed with 5 mL of trimethoprim–sulfamethoxazole twice daily for a week; **Conclusions:** This case highlights the rare involvement of *Acinetobacter lwoffii* in blistering distal dactylitis and emphasizes the importance of culture-guided diagnosis and treatment.

## 1. Introduction and Clinical Significance

Blistering distal dactylitis (BDD) was first explained and labeled by Hays and Mullard in 1972 [1]. It is a rare localized infection that typically involves the fat pad of the distal phalanx, most commonly affecting young children and adolescents. It is typically presented as superficial tense, tender, oval bullae with varying size filled with thin, seropurulent fluid. The primary etiologic agent is found to be Group A β-hemolytic *Streptococcus* with *Staphylococcus aureus* identified only in a minority of reported cases [2]. Although BDD is traditionally associated with Group A *Streptococcus* and *Staphylococcus aureus*, atypical organisms should be taken into account when treatment response is poor or clinical symptoms are uncommon. Since culture and susceptibility testing can show patterns of antimicrobial resistance and prompt change in empirical antibiotic therapy, the identification of rare organisms may have significant consequences for diagnostic evaluation and therapeutic management. Thus, by culture-guided selection of targeted antimicrobial drugs, microbiological confirmation not only improves patient treatment but also fosters antimicrobial stewardship [3,4].

In contrast, infections with atypical or opportunistic organisms are extremely rare. *Acinetobacter lwoffii* (AL) is an aerobic, non-fermentative, Gram-negative bacillus commonly colonizing the oropharynx and skin flora. Generally considered a low-virulence organism, it is recognized as an important opportunistic pathogen among immunocompromised patients [5]. A relatively small number of infections caused by *Acinetobacter lwoffii* have been reported, with most cases involving bloodstream infections or bacteremia associated with central intravascular catheters.

To our knowledge, this is the first documented case of Blistering distal dactylitis caused by *Acinetobacter lwoffii* in immunocompetent patients. Knowledge of this entity is crucial for early diagnosis and management.

## 2. Case Presentation

**Figure 1:**
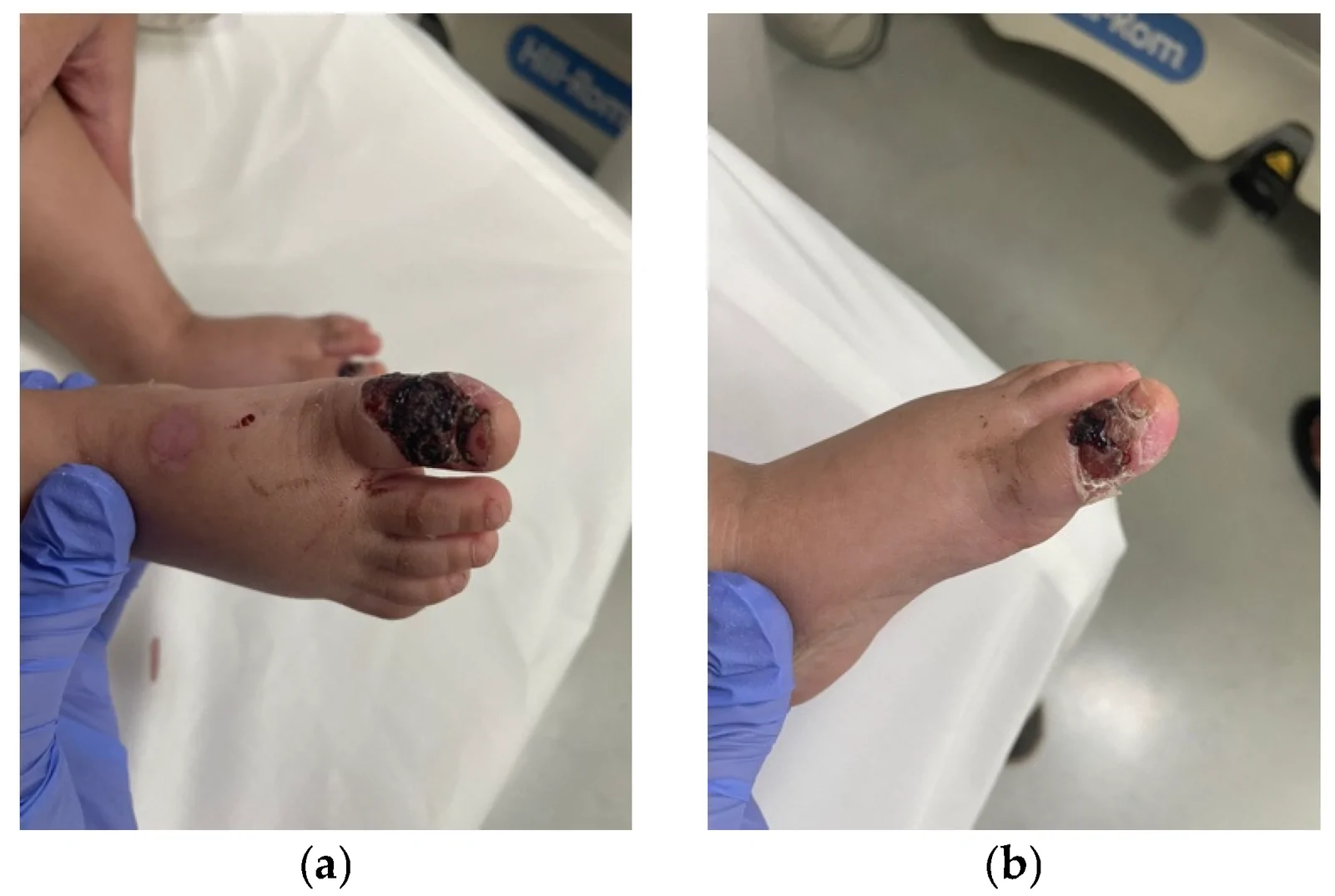
(**a**) Right great toe showing a well-defined hemorrhagic crusted erosion involving the distal nail unit and periungual skin with surrounding desquamation. (**b**) Left great toe showing a smaller well-defined hemorrhagic crusted periungual erosion with surrounding desquamation.

This is a case of an 18-month-old full-term boy, a product of spontaneous vaginal delivery, with vaccinations up to date and on formula milk. The patient is a known case of pulmonary stenosis that presented to the Emergency Department (ED) complaining of hemorrhagic crustation over the bilateral big toes and hemorrhagic crust over the right palm, which started 2 weeks ago as fluid-filled bullae over both great toes and then subsequently ruptured and evolved into hemorrhagic crusted lesions. The family denied any history of trauma, insect bites, use of new footwear, friction, daycare exposure, prior similar lesions, systemic symptoms such as fever, family history of immunodeficiency, or medication use at the time of presentation. Past medical history was only significant for pneumonia and hospital admission one month prior to ED presentation. On examination, the patient was vitally stable, active and in no distress. Upon dermatological examination, a single well-defined desquamating and hemorrhagic crusted erosion was found over the bilateral big toes (Figure 1). Then, a wound swab was obtained for bacterial culture, and blood cultures and laboratory investigations were collected. Hematological evaluation demonstrated unremarkable findings of complete blood counts, renal function test, liver function test, coagulation profile, and inflammatory markers along with neonatal profile. The following autoimmune marker and vasculitis workup were performed: Complement C3&4, Anti-Streptolysin O, and Anti-Nuclear Antibody (ANA), which were negative. Wound culture swab was taken in the ED and resulted in *Acinetobacter lwoffii* group, whereas the blood culture result was negative for growth in preliminary after 24 h and 5 days. Initial management included application of topical Fucidin and mupirocin ointment. Based on antibiotic sensitivity results and pediatric infectious disease team consultation, the recommendation was to initiate oral trimethoprim–sulfamethoxazole at a dose of 8 mg/kg/day divided into two daily doses. The prescribed suspension volume was 5 mL twice daily for 7 days. At the one-week follow-up visit in the dermatology clinic, the patient reported significant improvement, with complete resolution of the lesions. Wound culture swab was repeated which revealed no further growth. The patient was referred to the Allergy and Immunology clinic for assessment of possible immunodeficiency disorders; the evaluation was unremarkable, and no immunodeficiency was identified. No recurrence was observed at 4-week follow-up and the lesions healed without scarring. The parents reported satisfaction with treatment and relief after complete resolution.

## 3. Discussion

Blistering distal dactylitis (BDD) is a localized bacterial infection of the distal phalanx, characterized by tight bullae on an erythematous base. Although it is most common in children and teenagers, cases have been observed in infants and adults. BDD, first identified in 1972, is typically caused by Group A β-hemolytic *Streptococcus* or, less frequently, *Staphylococcus aureus*. The infection is superficial, affecting the epidermis and upper dermis, and is usually treated with simple drainage and suitable antibiotics [2]. In rare cases, opportunistic or Gram-negative microbes may be the cause [5]. Our patient’s case is remarkable as it represents, to our knowledge, the first report of *Acinetobacter lwoffii*-induced BDD in an immunocompetent Child. *A. lwoffii*, an aerobic, non-fermentative, Gram-negative coccobacillus, typically colonizes human skin and mucous membranes [3].

Although *A. lwoffii* is recognized as part of the normal skin and mucosal microbiota, several findings support its role as the causative pathogen in our patient rather than a contaminant. First, *A. lwoffii* was isolated from the wound culture obtained directly from the active lesion in the setting of compatible clinical findings. Second, the patient demonstrated complete clinical resolution following targeted antimicrobial therapy guided by susceptibility testing, accompanied by a subsequent negative culture. Third, alternative infectious and inflammatory causes were investigated and not supported by clinical, laboratory, or microbiological findings. Taken together, the temporal relationship between culture isolation, directed treatment, and clinical improvement strongly supports true infection rather than incidental colonization.

Despite being thought of as a low-virulence commensal, it can lead to opportunistic infections in patients who are hospitalized or have impaired immune systems [6]. But according to recent investigations, *A. lwoffii* can also cause community-acquired infections in healthy people, such as peritonitis and cellulitis [7,8]. Veraldi et al. described seven cases of BDD, all caused by *S. aureus*, supporting its emergence as the most frequent pathogen in both adults and children [9]. Compared with classic BDD caused by Group A β-hemolytic *Streptococcus*, our patient exhibited several unusual features. The lesions involved both great toes rather than a single digit, were associated with hemorrhagic crustation, and occurred in a very young child. These findings broaden the recognized clinical spectrum of BDD and suggest that atypical presentations may be associated with pathogens other than the traditionally implicated streptococcal organisms. Therefore, reliance solely on clinical appearance may be insufficient in atypical or bilateral cases.

Fretzayas et al. reported the first pediatric case of MRSA-associated BDD in a six-month-old infant, highlighting the need for culture confirmation before initiating therapy [10]. In contrast, our case involved *A. lwoffii*, a Gram-negative organism not previously associated with BDD, but the patient achieved complete recovery following a short course of oral trimethoprim–sulfamethoxazole selected on the basis of antimicrobial susceptibility testing and consultation with the pediatric infectious disease team. Because *Acinetobacter* species frequently demonstrate variable resistance profiles, culture-guided therapy is particularly important. The favorable response observed in this case supports the utility of susceptibility-directed treatment when uncommon pathogens are identified.

Although Group A β-hemolytic *Streptococcus* and *Staphylococcus aureus* remain the predominant causes of BDD, reports involving atypical organisms have gradually expanded the known microbiological spectrum of the disease. The identification of *A. lwoffii* in the present case further supports the possibility that uncommon pathogens may produce a clinical picture indistinguishable from classic BDD. Recognition of these atypical etiologies is clinically important because empirical antibiotic regimens may not provide adequate coverage and could delay recovery if microbiological confirmation is not obtained.

The exact source of infection remains uncertain. However, the patient’s hospitalization for pneumonia approximately one month before presentation may have facilitated transient colonization with *A. lwoffii*. Additional contributing factors may have included unrecognized minor trauma, local skin barrier disruption, moisture-related maceration, or environmental exposure, all of which have been proposed as facilitating factors in superficial bacterial infections. Nevertheless, no definitive predisposing factor was identified in this patient. Similar pathways have been hypothesized for streptococcal and staphylococcal BDD [8]. The thick stratum corneum of the distal phalanx can accumulate seropurulent fluid, resulting in tense bullae [8]. The antibiotics that have been demonstrated to effectively treat pediatric BDD cases with *S. aureus* or *Streptococcus pyogenes* infections are oral β-lactams, including cephalexin or amoxicillin [5,9]. The treatment of BDD caused by uncommon bacteria, such as *A. lwoffii*, necessitates tailored antibiotic therapy guided by culture results, as empiric treatment may fail. This case also highlights broader antimicrobial stewardship principles. Empirical treatment remains appropriate for most uncomplicated pediatric skin infections; however, atypical clinical features, bilateral involvement, treatment failure, or unusual lesion morphology should prompt bacterial culture and susceptibility testing. Early microbiological confirmation can minimize unnecessary antibiotic exposure, facilitate targeted therapy, and improve clinical outcomes. Microbiological confirmation is crucial for diagnosing BDD caused by *A. lwoffii* and selecting appropriate antibiotics. Misdiagnosis as bullous impetigo, herpetic whitlow, or contact dermatitis may delay treatment [10].

This report has several limitations. As a single case report, causal relationships cannot be definitively established, and the findings may not be generalizable to all patients with BDD. In addition, the precise route of acquisition of *A. lwoffii* could not be determined. Nevertheless, the microbiological findings, favorable response to targeted therapy, and absence of recurrence support the clinical significance of the isolate.

## 4. Conclusions

This case broadens the microbiologic range of BDD by demonstrating that even low-virulence organisms like *Acinetobacter lwoffii* can serve as real pathogens in immunocompetent children, emphasizing the need for acquiring cultures in atypical or nonresponsive presentations.

## Data Availability

Relevant data supporting the findings of this study can be made available upon reasonable request to the corresponding author.

## Abbreviations

The following abbreviations are used in this manuscript:

BDD	Blistering Distal Dactylitis
AL	*Acinetobacter lwoffii*
ANA	Anti-Nuclear Antibody
